# Is Regular Weight-Bearing Physical Activity Throughout the Lifecourse Associated with Better Bone Health in Late Adulthood?

**DOI:** 10.1007/s00223-022-00995-9

**Published:** 2022-06-17

**Authors:** Jean Zhang, Camille Parsons, Nicholas Fuggle, Kate A. Ward, Cyrus Cooper, Elaine Dennison

**Affiliations:** 1 MRC Lifecourse Epidemiology Centre Southampton General Hospital, Tremona Rd, Southampton, SO16 6YD UK; 2 NIHR Southampton Biomedical Research Centre, University of Southampton and University Hospital Southampton NHS Foundation Trust, Southampton, UK; 3 NIHR Musculoskeletal Biomedical Research Unit, University of Oxford, Oxford, UK; 4 Victoria University of Wellington, Wellington, New Zealand

**Keywords:** Weight bearing, Physical activity, Lifecourse, BMD

## Abstract

We considered how weight-bearing physical activity (WBPA) through the lifecourse related to bone health in late adulthood in the Hertfordshire Cohort Study (HCS), a cohort of community dwelling adults born 1931–9, to identify sex-specific differences and periods critical for optimal bone health. Available questionnaire data from 258 participants (128 men and 130 women) included current reported lifestyle factors (including physical activity) and WBPA, coded as participation in WBPA aged < 18 years; aged 18–29 years; aged 30–49 years and aged ≥ 50 years. Responses were recorded as none/once a month/once a week/> once a week. Hip bone mineral density (BMD) was measured using a Lunar Prodigy DXA scanner. The mean age was 75.4 (SD 2.5) years in men and 75.7 (SD 2.6) years in women. Men reported significantly higher levels of past WBPA aged < 18 years (*p* = 0.006) and aged 18–29 years than women (*p* < 0.001). We observed greater BMD at total hip in women who reported regular WBPA at ages 18–29 years (*p* = 0.02) and 30–49 years (*p* = 0.02) compared with those who reported no WBPA (*p* = 0.019), after adjustment for confounders including current activity levels. In this cohort of older adults, recalled regular WBPA around the time of peak bone mass acquisition was less common in women than men, but associated with higher hip BMD in women in late adulthood.

## Introduction

As a population, reduced physical activity is becoming increasingly common, and is associated with numerous adverse health consequences, including falls and fracture [1–3]. Physical inactivity was amongst the top 10 leading risk factors for disease burden in the 2010 Global Burden of Disease Study [4]. Recent data from World Health Organisation (WHO) highlight that 25% of adults and 80% of adolescents are insufficiently active [5], with current WHO guidance recommending that adults, including older people, undertake at least 150 min of moderate-intensity aerobic physical activity each week [5].

Previous studies have highlighted the benefits of weight-bearing activity for bone health [6–9] but much of this work is cross-sectional, with few studies describing activity profiles across the lifecourse, and associations with bone health in late adulthood. Studies involving children and adolescents have shown that regular participation in physical activity can have positive outcomes in bone structure in midlife [10, 11], whilst most research in older adults have shown those classified as active are more likely to experience a lower age-related decline in bone mineral density (BMD) [7, 8, 12], retain better balance and remain independent [13]. The few studies that have tracked physical activity from middle to later life have shown those who were active in their middle age were more likely to remain active in their older age [14]; that higher participation in physical activity was linked to better BMD [15] and lower bone loss [16]. Hence, few studies have considered relationships in both sexes, where lifecourse data are available.

In this study, we used a well-characterised longitudinal cohort of older adults to perform a lifecourse analysis of recalled weight-bearing physical activity (WBPA) and bone health. We sought to consider (1) whether higher physical activity at a particular time point was of benefit and (2) whether the same relationships were seen in men and women.

## Methods

Participants were recruited from the HCS [17], a study originally established to examine the relationship between growth in early life and adult diseases such as cardiovascular conditions and musculoskeletal diseases. The study design is described in detail elsewhere [17]. In brief, participants were born between 1931 and 1939, and were still currently living in Hertfordshire at the time of study. In this phase of the study, performed in 2011, 258 participants were recruited randomly selected from the total number, but all based in East Hertfordshire, 128 men and 130 women. Trained study nurses visited participants at home and administered questionnaires including, demographics, lifestyle and current physical activity levels. Lifestyle factors included smoking status (current or historical) and alcohol consumption (units consumed per week). Duration of current physical activity in the past 14 days was reported using the validated LASA Physical Activity Questionnaire (LAPAQ). Frequency and duration of activities over the past 2 weeks were recorded, including walking outside, cycling, gardening, a maximum of two sports and light/heavy household work. Anthropometry measurements including height and weight were measured, and Body Mass Index (BMI) was calculated. Height was measured to the nearest 0.001 m using a stadiometer. Weight was measured to the nearest 0.1 kg using a calibrated scale. Body mass index (BMI) was calculated as weight in kilograms divided by height in squared metres. Hip BMD was measured on participants using a Lunar Prodigy dual X-ray absorptiometry (DXA) scanner.

Personal recall of previous participation of WBPA was used in this study. Participants answered the following question: how often did you take part in sports and leisure time exercise involving weight-bearing activity? (e.g. running, racquet sports, football, rugby, hockey and dancing–not including walking, cycling or swimming) for each of four age groups. The age groups were as follows: < age 18 years, aged 18–29 years, ages 30–49 years and ≥ 50 years. Responses were categorised as none, once a month, once a week and more than once a week. The responses were scored as 0 for none, 1 for once a month, 2 for once a week and 3 for more than once a week to calculate a cumulative lifetime activity score.

The East and North Hertfordshire Ethical Committees granted approval for the study (10/HO311/590), and written informed consent was obtained from all participants.

### Statistical Analysis

Cohort characteristics were summarised using mean and standard deviations (SD), median and interquartile ranges (IQR) or frequencies and percentages (%) as appropriate. Differences between men and women were assessed using Student’s *t* tests, Mann–Whitney tests, χ2 tests or Fisher’s exact tests. The BMD variables were transformed using the Fisher–Yates rank-based inverse normal transformation to create *z* scores. Relationships between BMD in later life and the frequency of past WBPA were examined using linear regressions. Analyses were conducted with and without adjusting for age, BMI, social class, smoker status, alcohol consumption, current physical activity and dietary calcium intake, and, additionally, for years since menopause and HRT use in women. Data analysis was carried out using Stata statistical software (Statacorp, Texas, USA).

## Results

Data on past WBPA were available for 128 men and 130 women. The characteristics of these participants are summarised in Table 1. The mean age of participants was 75.4 (SD 2.5) years for men and 75.7 (SD 2.6) years for women. There were significantly more men who were current or ex-smokers than women (5.5% vs 0.8% and 56.3% vs 33.1%, respectively), and fewer men who had never smoked compared to women (38.3% vs 66.2%). Multimorbidity was common; 19.5% men and 28.5% women reported no co-morbidities; 39.1% men and 28.5% women reported 1 co-morbidity; 25.8% men and 23.1% women reported 2 co-morbidities, 8.6% men and 11.5% women reported 3 co-morbidities and lastly 7% men and 8.5% women reported ≥ 4 co-morbidities.

**Table 1:** Baseline characteristics of participants

	Men			Women			*p* value
	Total *N*	Mean	SD	Total *N*	Mean	SD	
Age (years)	128	75.4	2.5	130	75.7	2.6	0.27
Height (cm)	128	172.5	6.7	129	159.6	5.9	< 0.01
Weight (kg)	128	81.5	11.4	130	72.5	13.2	< 0.01
BMI (kg/m^2^)	128	27.4	3.6	129	28.4	4.9	0.06

	Total *N*	Median	IQR	Total *N*	Median	IQR	*p* value
Daily dietary calcium intake (mg)	128	1237	(1005–1418)	130	1118	(941–1281)	0.01
Alcohol consumption (units per week)	128	7	(1.8–14.3)	130	0.8	(0.0–5.0)	< 0.01

	Total *N*	*n*	%	Total *N*	*n*	%	*p* value
Smoker status	128			130			
Never		49	38.3		86	66.2	< 0.01
Ex/Current		79	61.7		44	33.9	
Social class	122			130			
I-IIINM		56	45.9		68	52.3	0.31
IIIM-V		66	54.1		62	47.7	

There was a modest difference between men and women’s current physical activity at the time of study (median (IQR) activity time 194 (110–298) minutes per day for men, 206 (146–277) minutes per day for women). There were, however, statistically significant differences between men and women in the frequency of WBPA over the lifecourse (Table 2). Men reported a higher frequency of weight-bearing physical activity during their younger years with 53.4% of men reporting been active more than once a week up to 18 years of age, and 41.6% reporting being active more than once a week when aged 18–29, compared to 30.3% (*p* = 0.006) and 15.6% (*p* < 0.001) of women, respectively. For both men and women, the frequency of physical activity decreased as their age increased and sex differences became less marked; in those aged 30–49 years, 23.7% men and 12.5% of women reported activity more than once a week (*p* = 0.18). The rate decreased further in those aged 50 years or older, with 16.2% of men and only 8.5% of women performing WBPA more than once a week (*p* = 0.102).

**Table 2:** Past weight-bearing physical activity

	Men			Women			*p* value
	Total, *n*	*n*	%	Total, *n*	*n*	%	
Weight-bearing activity up to age 18	118			109			0.006
None		19	16.1		25	22.9	
Once a month		7	5.9		10	9.2	
Once a week		29	24.6		41	37.6	
More than once a week		63	53.4		33	30.3	
Weight-bearing activity aged 18–29	113			109			< 0.001
None		19	16.8		26	23.9	
Once a month		13	11.5		29	26.6	
Once a week		34	30.1		37	33.9	
More than once a week		47	41.6		17	15.6	
Weight-bearing activity aged 30–49	114			112			
None		36	31.6		43	38.4	0.177
Once a month		22	19.3		25	22.3	
Once a week		29	25.4		30	26.8	
More than once a week		27	23.7		14	12.5	
Weight-bearing activity aged over 50	117			118			0.102
None		64	54.7		59	50.0	
Once a month		18	15.4		22	18.6	
Once a week		16	13.7		27	22.9	
More than once a week		19	16.2		10	8.5	

**Fig. 1:**
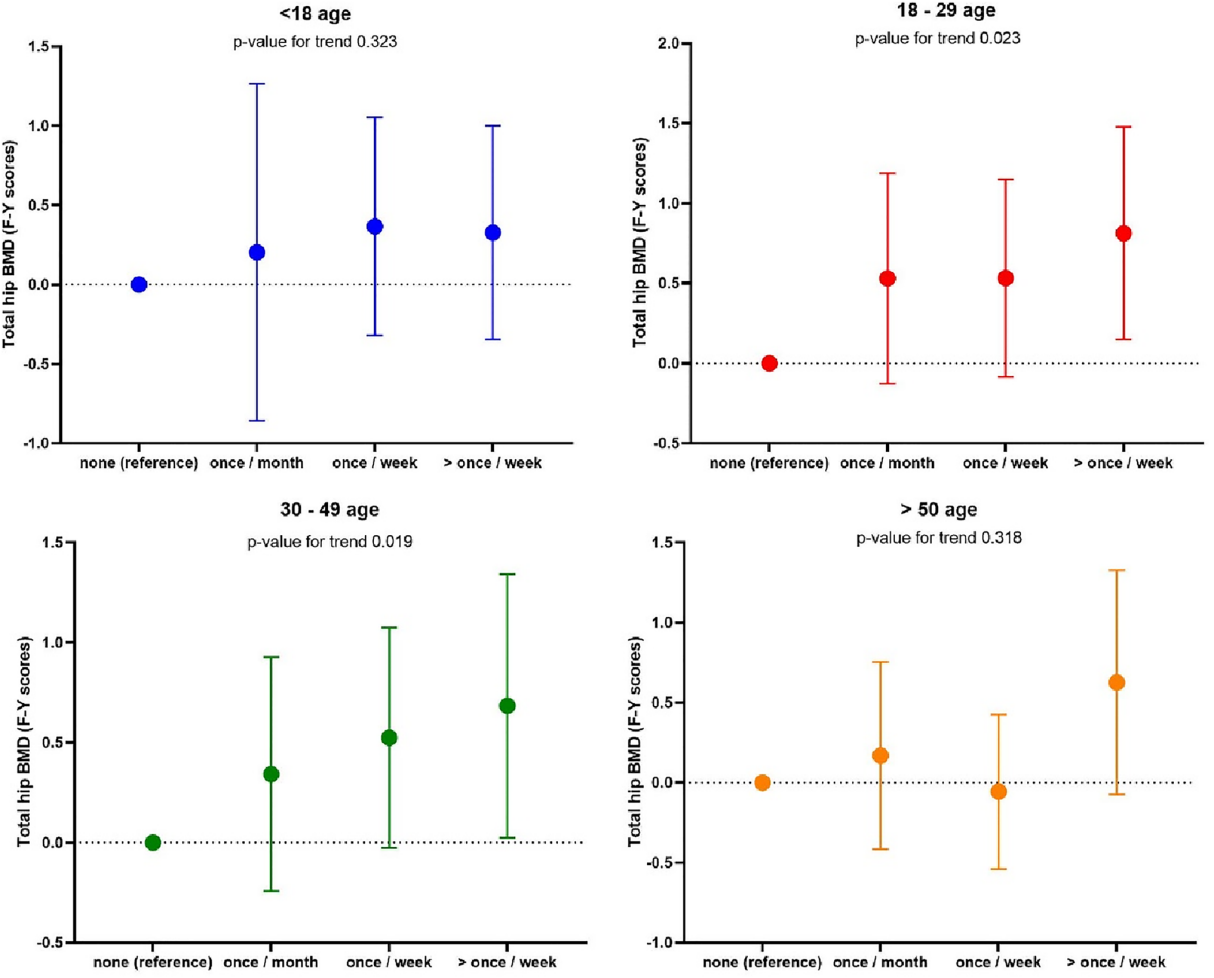
Regression coefficients (and 95% CIs) for the associations between past WBPA at different ages and total hip BMD in women. Adjusted for age, BMI, social class, smoker status, alcohol consumption, current physical activity, dietary calcium intake and for years since menopause and HRT use

**Fig. 2:**
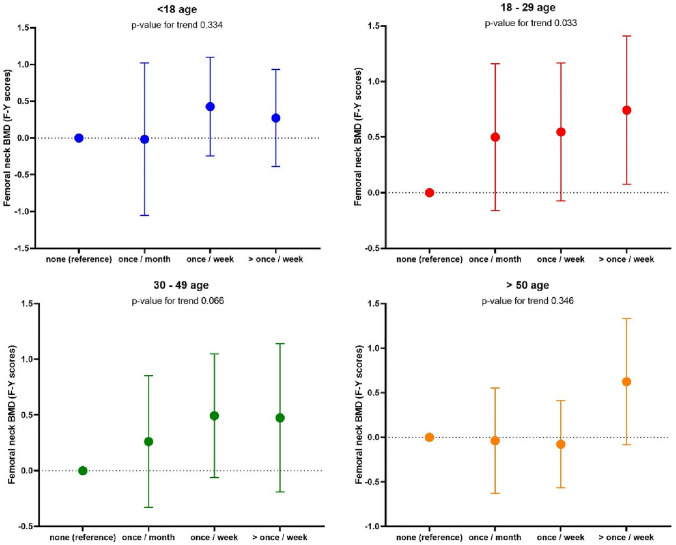
Regression coefficients (and 95% CIs) for the associations between past WBPA at different ages and femoral neck BMD in women. Adjusted for age, BMI, social class, smoker status, alcohol consumption, current physical activity, dietary calcium intake and for years since menopause and HRT use

**Fig. 3:**
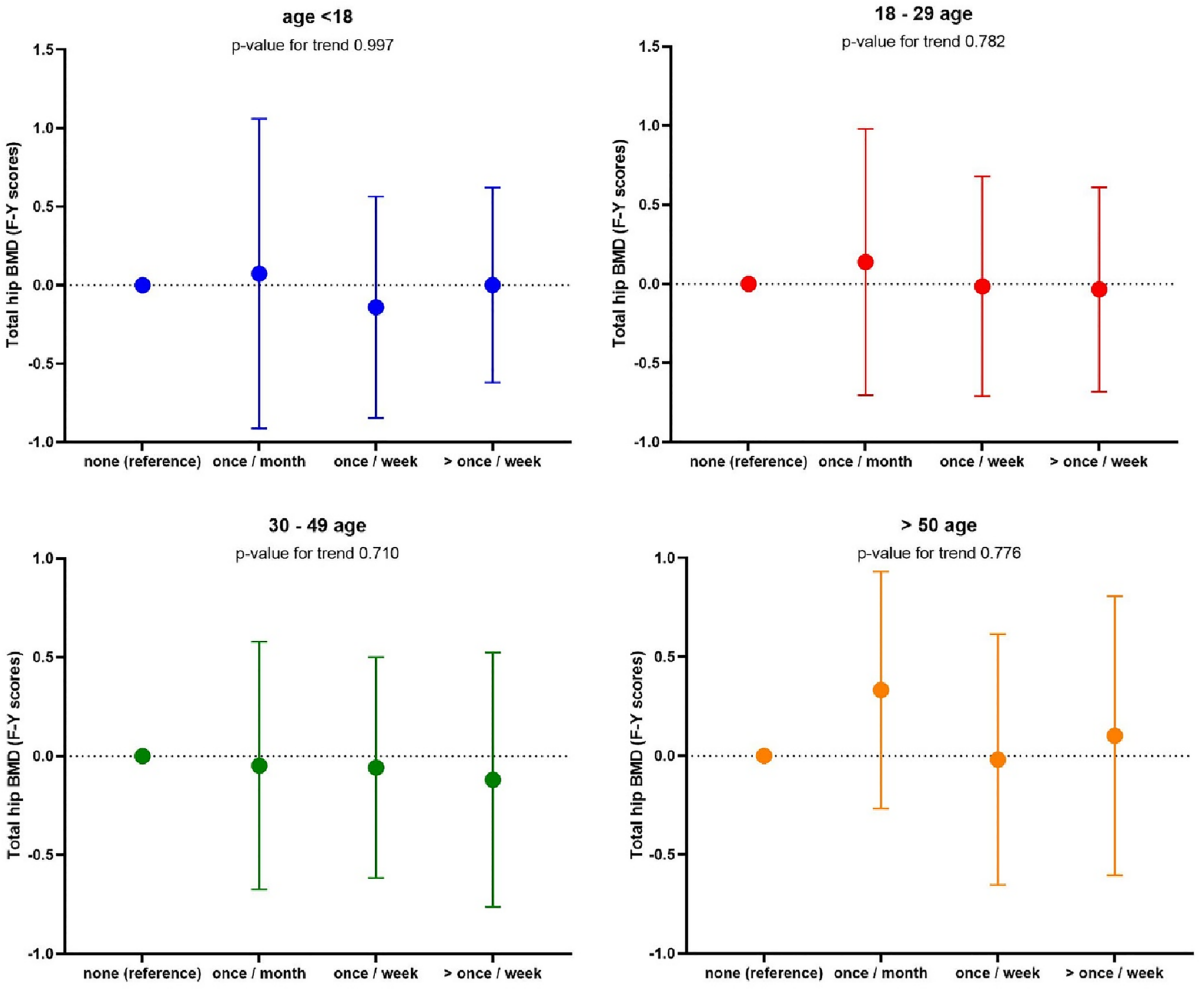
Regression coefficients (and 95% CIs) for the associations between past WBPA at different ages and total hip BMD in men. Adjusted for age, BMI, social class, smoker status, alcohol consumption, current physical activity and dietary calcium intake

**Fig. 4:**
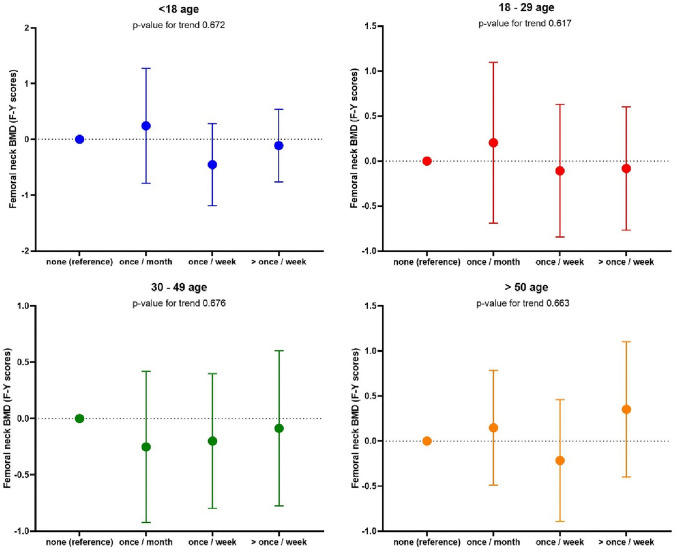
Regression coefficients (and 95% CIs) for the associations between past WBPA at different ages and femoral neck BMD in men. Adjusted for age, BMI, social class, smoker status, alcohol consumption, current physical activity and dietary calcium intake

We next considered relationships between WBPA and BMD. No relationship was observed between WBPA and BMD in men at any timepoint. Relationships are shown in Figs. 1, 2, 3, 4. We observed an association between BMD at the total hip and WBPA at ages 18–29 years (regression coefficient (*β*) exercise weekly 0.72 *z* score (95% confidence interval (CI) 0.13, 1.31), *p* = 0.02; *β* exercise more than once a week 0.83 *z* score (95% CI 0.20, 1.46), *p* = 0.01), when adjusted for age, BMI, social class, smoker status, alcohol consumption, current physical activity, dietary calcium intake, years since menopause and HRT use, compared to those reporting no WBPA (Fig. 1). Similarly, there was also an association between total hip BMD and WBPA at ages 30–49 years (*β* exercise weekly 0.52 *z* score (95% CI 0.02, 1.02), *p* = 0.04; *β* exercise more than once a week 0.78 *z* score (95% CI 0.15, 1.41), *p* = 0.02), when adjusted for confounders. Furthermore, in women, there was a significant dose response between BMD at the total hip and WBPA at ages 18–29, *p* 0.023, and ages 30–49, *p* 0.019 when compared to those reported no activity (Fig. 1). A similar trend was also noted between BMD at femoral neck and WBPA, with ages 18–29 reaching statistical significance, *p* 0.033 when compared to those reported no activity (Fig. 2). In men, no such relationship was observed at total hip BMD (Fig. 3) nor femoral neck BMD (Fig. 4). A cumulative activity score was calculated to assess the impact of partaking WBPA across the lifecourse. A cut-off point of 8 was utilised, as on average the participant would have to partake in WBPA once a week or more than once a week at every time point; 65 (37.8%) achieved this threshold. Whilst the mean total hip BMD differences were not statistically significant (*p* = 0.46), more regular PA through the lifecourse was associated with higher BMD.

## Discussion

In this study, we have examined the relationship between recalled WBPA over the lifecourse and bone health in late adulthood. Men in our cohort group reported higher rates of participation in WBPA from 18 to 29 years, compared to women. However, the overall frequency of WBPA decreased as age increased in both sexes, and differences in WBPA between men and women became less pronounced with age. Indeed, the median time of current physical activity in older age was slightly higher in women than men, which may reflect the inclusion of household tasks in the LAPAQ questionnaire. We observed relationships between WBPA in women at the time of peak bone mass acquisition and BMD measured at later age, with a positive dose-related relationship between higher frequency of past self-reported WBPA at the age of bone acquisition observed and BMD at total hip and femoral neck in women. These relationships were robust to adjustment for current WBPA. This relationship was not seen in men.

There are of course limitations to our study; our cohort is based on a group of men and women recruited because they were born in Hertfordshire and still lived there in adult life. However, we have previously shown this group to be representative of the general UK population with regard to lifestyle characteristics such as body mass index and smoking [18]. Our sample size is modest, but perhaps the most significant limitation is the use of self-reported WBPA earlier in life, although current PA was validated in a subset of our cohort [19]. However, unless differential recall bias is operating, this should not affect the validity of our results. The type of physical activity asked in the questionnaire is weight-bearing physical activity, and therefore, there was no differentiation between sports-related or leisure-related activity. We also did not consider occupational activity specifically, although this may be less relevant in this cohort of women born in the 1930s.

In our study, we see a gender difference in physical activity participation. We hypothesise during the 1940s–1960s women will have a different physical activity profile compared to men. Several studies of nationally representative samples have shown men are more likely to participate in moderate-vigorous physical activity than women when younger [20–22]. However, women spend less time been sedentary than men in older adulthood [21, 22], and we observed the median time spent been active was higher for women than men. Previous work has highlighted the importance of peak bone mass in the risk of later osteoporosis [23] and our data reinforce this. In addition, recent data showed moderate to vigorous-intensity activity at adolescents (ages 12, 14 and 16) was associated with higher femur BMD, whereas light-intensity activity did not derived such benefit [24]. Whilst we did not observe strong relationships between being active throughout the 4 timepoints across the lifecourse, this may reflect small numbers of participants who reported regular weight-bearing activity, hence limiting our ability to consider this. Other studies have suggested that being active throughout the lifecourse is associated with better bone health later in life and the trends that we did observe were in accord with this [25, 26].

In line with the 1946 British Birth Cohort, we note a strong positive relationship between past WBPA at ages 18–29 and ages 30–49 and hip BMD at older adulthood in women; however, in the 1946 British Birth Cohort, the association was stronger in men than women [15]. This may reflect methodological differences between the two studies including the type of physical activity data collected. Specifically in the 1946 British Birth Cohort, researchers collected data on all leisure time physical activity, whereas our study focussed on WBPA only. In addition, although our own sample size was modest compared to the 1946 British Birth Cohort, we were able to consider WBPA at earlier time points from < 18 years compared with the 1946 British Birth Cohort which started at age 36. In other work, however, several authors have reported associations with WBPA and bone health in women consistent with our own findings [27, 28]. In the Northern Finnish cohort, high level of past physical activity over the lifecourse from 14 to 46 years of age was associated with larger vertebral cross-sectional area in women, whereas this association was not observed in men [28]. Finally, in recent work, in the Tromsø cohort, it was suggested the relationship between WBPA, and bone health may differ in male and female adolescents, with girls reporting high levels of PA having the best bone profiles [29].

In conclusion, we have highlighted differences in WBPA across the lifecourse using a historical cohort and reported a positive dose-related association between frequency of past WBPA and BMD in later life in women. This suggests we should encourage young women to participate in regular WBPA throughout the lifecourse, but particularly at the time of peak bone mass accrual, to reap the benefits for bone health later in life.
